# Ontario Veterinary Association

**Published:** 1893-01

**Authors:** 


*Ontario Veterinary Association.*—The annual meeting of the Ontario Veterinary Association was held in the Ontario Veterinary College, Toronto, Canada, on Thursday, December 22d.

Mr. MacArthur, President, in his opening address, urged on graduates to attend the meetings of the Association, dwelling on their value as cultivating a kindly feeling generally amongst members, and the benefit mutually derived from an interchange of ideas.

Amongst the subjects brought forward, the much disputed cattle-dehorning question was discussed, on which Professor Smith made a few remarks, he having been a member of the Government Commission appointed to investigate.

A resolution was passed that the Association should meet in London, Ontario, in June, 1893.

The officers elected for the ensuing year are: Messrs. John Wende, V.S., Buffalo, N. Y., U. S., President; W. H. Burns, V.S., King, Ontario, First Vice-President; G. L. Robson, V.S., Manchester, Ontario, Second Vice-President; C. H. Sweetapple, V.S., Toronto, Ontario, Secretary; W. Cowan, V.S., Galt, Ontario, Treasurer. Messrs. F. J. Gallanough, V.S., D. Hamilton, V.S., H. Butcher, V.S., W. C. Hand, V.S., J. F. Quinn, V.S., W. Gibb, V.S., W. J. Wilson, V.S., and H. Hopkins, V.S., Directors. Messrs. O'Neil and Elliott, Auditors.

Messrs. Wilson, Sr., and O'Neil were appointed to represent the Association at the Western Fair Association. Mr. Cowan was appointed Delegate to the Central Farmers' Institute. Professor Smith, Dr. Duncan and the President were appointed to represent the Association at the International Veterinary Congress to be held in Chicago. Messrs. John Wende, W. Cowan and W. Gibb were appointed to read papers at the meeting to be held in London.

A unanimous vote of thanks was tendered to Mr. MacArthur, the retiring President, for his able conduct during his term of office.

